# Protective Effect of Ischemic Preconditioning on Myocardium Against Remote Tissue Injury Following Transient Focal Cerebral Ischemia in Diabetic Rats

**DOI:** 10.5935/abc.20170164

**Published:** 2017-12

**Authors:** Meltem Kumas, Ozge Altintas, Ersin Karatas, Abdurrahim Kocyigit

**Affiliations:** 1 BezmiAlem Vakif University - Vocational School of Health Services - Medical Laboratory Techniques - Turquia; 2 Kirklareli State Hospital, Neurology Clinic - Turquia; 3 Gebze Technical University, Department of Molecular Biology and Genetics- Turquia; 4 Bezmialem Vakif University - Medical Faculty - Medical Biochemistry Department - Turquia

**Keywords:** Ischemic Preconditioning Myocardial, Middle Cerebral Artery, Rats, Diabetes Mellitus, Experimental

## Abstract

**Background:**

Remote ischemic preconditioning (IPreC) could provide tissue-protective effect at a remote site by anti-inflammatory, neuronal, and humoral signaling pathways.

**Objectives:**

The aim of the study was to investigate the possible protective effects of remote IPreC on myocardium after transient middle cerebral artery occlusion (MCAo) in streptozotocin- induced diabetic (STZ) and non-diabetic rats.

**Methods:**

48 male Spraque Dawley rats were divided into eight groups: Sham, STZ, IPreC, MCAo, IPreC+MCAo, STZ+IPreC, STZ+MCAo and STZ+IPreC+MCAo groups. We induced transient MCAo seven days after STZ-induced diabetes, and performed IPreC 72 hours before transient MCAo. Remote myocardial injury was investigated histopathologically. Bax, Bcl2 and caspase-3 protein levels were measured by Western blot analysis. Total antioxidant status (TAS), total oxidant status (TOS) of myocardial tissue were measured by colorimetric assay. Oxidative stress index(OSI) was calculated as TOS-to-TAS ratio. For all statistical analysis, p values < 0.05 were considered significant.

**Results:**

We observed serious damage including necrosis, congestion and mononuclear cell infiltration in myocardial tissue of the diabetic and ischemic groups. In these groups TOS and OSI levels were significantly higher; TAS levels were lower than those of IPreC related groups (p < 0.05). IPreC had markedly improved histopathological alterations and increased TAS levels in IPreC+MCAo and STZ+IPreC+MCAo compared to MCAo and STZ+MCAo groups (p < 0.05). In non-diabetic rats, MCAo activated apoptotic cell death via increasing Bax/Bcl2 ratio and caspase-3 levels. IPreC reduced apoptotic cell death by suppressing pro-apoptotic proteins. Diabetes markedly increased apoptotic protein levels and the effect did not reversed by IPreC.

**Conclusions:**

We could suggest that IPreC attenuates myocardial injury via ameliorating histological findings, activating antioxidant mechanisms, and inducing antiapoptotic activity in diabetic rats.

## Introduction

Ischemic preconditioning (IPreC) has been described to reduce the ischemia- reperfusion injury by triggering transient, brief episodes of ischemia to target organs. IPreC can be induced locally when the preconditioning stimulus is applied to the same tissue or can be a protection at distant tissues, a phenomenon known as remote IPreC (rIPreC).^1^ The rIPreC can primarily apply to a target organ, but if a brief ischemia is induced in non-target tissue, confers protection at a remote site such as the brain, non-infarcted adjacent myocardial tissue, lung, kidney, intestine, or skeletal muscle. rIPreC produces a similar degree of tissue protection as IPreC^1-5^ does. Recent studies indicated that brief ischemia-reperfusion induced in further tissue could provide tissue-protective effect at a remote site by anti-inflammatory, neuronal, and humoral signaling pathways.^1,6^

Several studies have shown that hyperglycemia causes endothelial dysfunction in the blood-barriers and diabetic cardiomyopathy.^7-9^ Clinical studies have demonstrated that hyperglycemia increased the size of ischemic infarct area and caused poor clinical outcome after stroke.^10^

Oxidative stress is a principal parameter for evaluating ischemia/reperfusion injuries in patients with diabetes mellitus.^11-12^ Myocardial cells also undergo death in response to hyperglycemia and ischemia/reperfusion injury.^13^ In the current study, we postulated that there is a possible remote effect of cerebral ischemic preconditioning on the myocardium.

The current database shows the connection between heart disease and acute stroke. Cardiac arrhythmia, myocardial dysfunction and serum cardiac enzyme elevation are known to develop after acute stroke onset.^14^ Acute ischemic cerebrovascular events can induce various myocardial changes. The cardiac lesions are not seen earlier than six hours after the acute cerebral event, nor later than two weeks, cardiac injury occur as a result of intense activation of the sympathetic nervous system.^15^

Diabetes is an important modifiable risk factor for stroke, especially ischemic strokes. In this study, we hypothesized that ischemic preconditioning could play a crucial role on cardio-neuroprotection, which was triggered by hyperglycemia. Therefore, we aimed to evaluate the effect of remote ischemic preconditioning on myocardial tissue after transient intraluminal middle cerebral ischemia reperfusion injury in diabetic and non-diabetic rats.

## Methods

### Animals

All animals were obtained from the Experimental Animal Research Laboratory at Bezmialem Vakif University, Istanbul, Turkey. Animals had free access to food and water at controlled room temperature (22-25°C) under a 12:12-h day/night cycle for the duration of the study. During the surgical procedures, body temperature was monitored using a Nimomed® infrared thermometer.

### Induction of experimental diabetes mellitus by Streptozotocin in rats

Streptozotocin (STZ) induces diabetes within 3 days by destroying the pancreatic beta cells.^16,17^ Streptozotocin solution (STZ, Sigma Chemical Corp., Germany) was prepared in 0.1mol/L citrate buffer, pH 4.5, immediately before use. Diabetes was induced in rats by a single injection of STZ at the dose of 50 mg/kg of the body weight intraperitoneally and they were fed normally thereafter. Insulin was not administered. The other group of animals received an equal volume of saline solution. Blood glucose concentrations were monitored before STZ injection, at the 6^th^ hour after STZ injection and on the 3^rd^ day after STZ injection using an ACCU-CHEK^®^(Active Glucometer, Roche Diagnostics GmbH, Germany). The blood samples were collected from the rat’s dorsal pedal veins. Rats with a glucose concentration exceeding 300 mg/dL were considered diabetic.^18,19^ All rats became diabetic after STZ injection. According to published experimental research, about six hours later, hypoglycemia occurs with high levels of blood insulin. ^11^ In order to counteract the initial hypoglycemia after STZ injection, we included the option for rats to take 10% dextrose in drinking water supplied to them approximately 6 hours post STZ injection, in addition to their normal water, for the first 24 hours.

### Ischemic preconditioning (IPreC)

Ischemic preconditioning (IPreC) involved three cycles of 10 minutes of reperfusion and 10 minutes of occlusion of unilateral-left proximal internal carotid artery.^5^ IPreC was performed 72 hours before transient MCAo and 4-days after on STZ-induced diabetic rats. The animals were anesthetized with ketamine (4 mg/100 g) and xylazine (1.5 mg/100 g) by intramuscular injection and placed on an operation plate in the supine position. Their heads and limbs were fixed. After shaving and sterilization, a cervical median incision (3-4 cm long) was made. Precervical fascia and muscle were isolated with forceps, and fascia and muscle on the inside of the sternocleidomastoid were dissociated. Arterial pulses were visible. Tissues surrounding the artery were carefully dissociated, without injury to the vagus nerve. The left common carotid artery and the left external carotid artery were exposed through a midline neck incision. First, the left internal carotid artery (ICA) was occluded by a micro clamp. Then, the micro clamp was removed to restore blood flow after 10 minutes of reperfusion, followed by 10 minutes of occlusion. After removing the micro clamp, we observed that the left ICA was reinjected anterogradely. Sham-surgery controls were operated with the same procedures without artery occlusion.

### Middle cerebral artery occlusion (MCAo)

The most common stroke model, due to its relevance to human stroke, is focal MCAo.^20,21^ In the present study, we induced a 3-hour transient proximal MCAo followed by 3-hour of reperfusion to cause remote ischemia-reperfusion injury to measure the level of oxidative stress markers to verify whether they were associated with myocardial tissue damage and the possible protective effect of ischemic preconditioning. Focal cerebral ischemia was induced using an endovascular middle cerebral arterial occlusion technique, as described previously.^20,21^ The sham operation consisted of the same manipulation but without introduction of the monofilament.

### Study design

Power analysis was used to estimate sample size. The sample size was calculated to be forty-eight with a 3% margin of error at a significance level of 0.05 for the 80% power value (Type I error = 0.05; statistical power = 0.80). Forty-eight male Sprague-Dawley rats (450-500 g; 10-12 months old) were divided into eight groups; Sham operated group (n = 6), STZ-induced diabetic (STZ) rat group (n = 6), MCAo group (n = 6), Ischemic-preconditioning (IPreC) group (n = 6), Ischemic-preconditioning (IPreC) + MCAo group (n = 6), Diabetic (STZ) Ischemic-preconditioning (IPreC) group (n = 6), Diabetic (STZ) MCAo group (n = 6) and Diabetic (STZ) Ischemic-preconditioning (IPreC) + MCAo group (n = 6).

A 3-hour transient proximal MCAo was induced in the experiment groups (MCAo, IPreC+ MCAo, STZ+ MCAo, STZ+ IPreC+ MCAo). We induced transient MCAo seven days after STZ-induced diabetes and performed IPreC 72 hours before transient MCAo to assess whether IPreC could have a protective effect on the remote tissue. All animals were sacrificed at 6^th^ hour after 3-hour of occlusion followed by 3-hour of reperfusion. From each rat brain, total hemispheric infarct volumes were evaluated in coronal brain specimens due to analyze,^2,3,5^ triphenyltetrozolium chloride (TTC) - staining changes on basal ganglia and cortex, which are the localizations of the ischemic core in this model. In addition, all animals were weighed every day during the study period using a digital scale. Blood glucose concentrations were monitored before STZ injection, at the 6^th^ hour after STZ injection, on the 3^rd^ day after STZ injection and after the rats were sacrificed.

### Assessment of infarct volume

Infarct volumes were calculated using TTC- stained brain sections, as described previously^5^. After the sacrifice, the brains were removed inmediately, and cut into 2-mm coronal sections. Samples were then incubated for 30 min in a 2% solution of TTC at 37°C and fixed by immersion in 10% buffered formalin solution. Five brain sections per animal were stained with TTC and then photographed. Cerebral infarct volumes were assessed by using the image analysis program of Adobe Photoshop CS5 extended (version 12.1).

### Tissue homogenization

Left ventricle was cut up into appropriately small pieces for analysis (300mg) and placed into microcentrifuge tubes than washed 3x with 1ml PBS and aspirated. Stainless steel beads (1.6mm blend) used for homogenization with NP-40 lysis buffer (2 mM Tris-Cl pH 7.5, 150 mM NaCl, 10% glycerol and 0.2% NP-40 plus a protease inhibitor cocktail). After homogenization, homogenates were centrifuged at 19.700 x *g* for 30 minutes at +4ºC. Supernatant was used as protein samples.

### Measurement of total oxidant status

Myocardial TOS level was measured using a novel automated method developed by Erel.^22^ Oxidants present in a sample oxidize the ferrous ion of an o-dianisidine complex to ferric ion. Oxidation is enhanced by glycerol, which is abundant in the reaction medium, and the ferric ion forms a colored complex with xylenol orange under acidic conditions. Color intensity, which can be measured spectrophotometrically, is associated with the total level of oxidants present. Hydrogen peroxide is used to calibrate the assay and results are expressed in terms of micromoles of hydrogen peroxide equivalent per liter (mmol H_2_O_2_ equiv. /l).

### Measurement of total antioxidant status

Myocardial TAS level was measured using another novel automated method developed by Erel.^23^ It involves production of the hydroxyl radical, which is a potent biological reactant. A ferrous ion solution (Reagent 1) is mixed with hydrogen peroxide (Reagent 2). Radicals produced by the hydroxyl radical, including the brown dianisidinyl radical cation, are also potent in biological terms. Thus, it is possible to measure the antioxidative capacity of a sample in terms of inhibition of free radical reactions initiated by production of the hydroxyl radical. Variation in assay data is very low (less than 3%) and results are expressed as mmol Trolox equiv./l. Results were given for 1mg total protein in tissue.

### Measurement of oxidative stress index (OSI)

The OSI level was the myocardial TOS-to-myocardial TAS ratio, but TAS values were changed to mmol/l. Each OSI was calculated as follows: OSI (arbitrary units) = TOS (mmol H_2_O_2_/l)/TAS (mmol Trolox/l).^24^

### Histopathological analysis

Left ventricle was also evaluated for histopathological analysis. Sections were stained with hematoxylin-eosin and Masson’s trichrome methods. We used hematoxylin-eosin stain for routine detection of pathological alterations including necrosis, congestion, and infiltration. Masson’s trichrome method was chosen for determining fibrosis on myocardium if any. Sections were examined and scored by an observer who was blind to the identification of the groups using a Nikon Eclipse i5 light microscope with a Nikon DS-Fi1c camera, and Nikon NIS Elements version 4.0 image analysis systems (Nikon Instruments Inc., Tokyo, Japan). Myocardial damage was scored in terms of necrosis, congestion, and mononuclear cell infiltration. Each data was scored as: 0: absent, 1: minimal, 2: moderate, 3: severe damage.

### Western Blot

Total membrane protein was extracted from the homogenized tissue samples as follows. Heart tissues of all groups were homogenized in lysis buffer (2 mM Tris-Cl pH 7.5, 150 mM NaCl, 10 % glycerol and 0.2% NP-40 plus protease inhibitor cocktail) for 30 min on ice. Then homogenates were centrifuged, (Beckman Coulter, Krefeld, Germany) at 19.700 x *g* for 10 min at 4°C, and the final supernatant was used as the total membrane protein. Gel samples were made by adding 100 µl Laemmli sample buffer containing 2% SDS (Santa Cruz, Paso Robles, CA) to a 10 mg total protein. The protein concentration was measured using Bradford method 40 micrograms of total protein from each sample was loaded onto 8%-12% sodium dodecyl sulfate-polyacrylamide electrophoresis (PAGE) gel for separation. The separated protein was transferred to a polyvinylidene difluoride (PVDF) membrane (Millipore, Billerica, MA). After incubation in 5% skim milk for 2 h at room temperature to block nonspecific binding, the PVDF membrane was reacted for 16 h at 4°C. The rabbit anti-rat caspase 3, Bax and Bcl-2 monoclonal antibodies were purchased from Sigma-Aldrich (St. Louis, MO, USA). All antibodies diluted 1:1000 in tris-buffered saline plus Tween 20 (TBST: 20 mM Tris HCl, 137 mM NaCl and 0.1% Tween-20, pH 7.6) containing 5% skim milk powder. The membrane then was washed with tris-buffered saline plus Tween (TBST: 20 mM Tris HCl, 137 mM NaCl and 0.1% Tween-20, pH 7.6) three times for 10 min each time and incubated with horseradish peroxidase (HRP) labeled anti-rabbit IgG antibodies diluted 1:5000 in TBS containing 5% skim milk powder (Santa Cruz). Finally, the PVDF membrane was washed three times with TBST for 10 min each time, reacted with Pierce ECL Western blotting substrate (Thermo Scientific) and visualized using the Fussion Fx7 Imaging System (Vilber Lourmat SA, France). The b-actin antibody (Santa Cruz) was used as a loading control. For semiquantitative analysis, the grayscales of Caspase-3, Bax, Bcl-2 and b-actin bands were measured using Image J software. The ratio of Caspase 3, Bax, Bcl-2 to b-actin were calculated.

### Statistical analysis

Normality of the all data was tested with Kolmogorov-Smirnov D test. Since they were normally distributed (Kolmogorov-Smirnov D test, p ≥ 0.05), parametric test ANOVA (post-hoc: Tukey’s HSD) was used for multiple comparisons. Continuous measurements are expressed as mean and standard deviation (mean ± 2SD) for each group. P values < 0.05 were considered statistically significant. All statistical analyses and bar charts were done with SPSS 20.0 (IBM, New York, USA), MS Office Excel, and Graph Pad Prism 6.

## Results

### Assessment of oxidative stress parameters

The lowest mean myocardial TAS value was detected in 0.96 ± 0.15 (mean ± 2SD) STZ+ MCAo group, whereas the highest values were measured as 1.58 ± 0.56 and 1.57 ± 0.88 in sham and IPreC+ MCAo groups, respectively. Inducing ischemia reperfusion injury significantly decreased myocardial TAS in all related groups when compared to sham and IPreC groups (p = 0.003 and p = 0.042, respectively.). Moreover, ischemic preconditioning significantly increased the mean myocardial TAS value after ischemia- reperfusion injury in non-diabetic rats (IPreC+ MCAo vs. MCAo (1.07 ± 0.30), p = 0.008), whereas the protective effect did not appear in diabetic rats (STZ+ MCAo+ IPreC (1.13 ± 0.50) vs. STZ+ MCAo (0.96 ± 0.30), p > 0.05).

The mean myocardial TOS levels of STZ and MCAo groups were 12.79 ± 1.12 and 12.74 ± 1.54, respectively. Thus, those levels of IPreC (11.17 ± 1.26) and sham (11.05 ± 1.56) groups were similar. The result could indicate that ischemic preconditioning does not exceed the threshold for tissue damage. Besides, a clinical study was reported that diabetes might prevent ischemic preconditioning.^25^ In contrast, we found that ischemic preconditioning lowered the oxidant capacity in diabetic rats (STZ vs. IPreC+ STZ (11.62 ± 1.74), p = 0.036). Similarly, IPreC significantly reduced the mean myocardial TOS level in both diabetic and non-diabetic rats which underwent middle cerebral artery occlusion (MCAo vs. IPreC+ MCAo (10.96 ± 1.72), p < 0.001; STZ+ MCAo (12.81±1.46) vs. STZ+ MCAo+ IPreC (12.33 ± 0.58), p = 0.04).

In all study groups, the highest OSI value, which is determined based on myocardial TOS/TAS ratio, were detected as 12.15 ± 4.26 and 13.61 ± 5.28 in MCAo and STZ+ MCAo groups, respectively. Both diabetic and non-diabetic rats that were induced ischemia-reperfusion injury, significantly demonstrated lower OSI values following IPreC, in comparison with non-IPreC related ones (MCAo vs. IPreC+ MCAo, p= 0.005 and STZ+ MCAo vs. STZ+ IPreC+ MCAo, p = 0.037).

**Figure 1:**
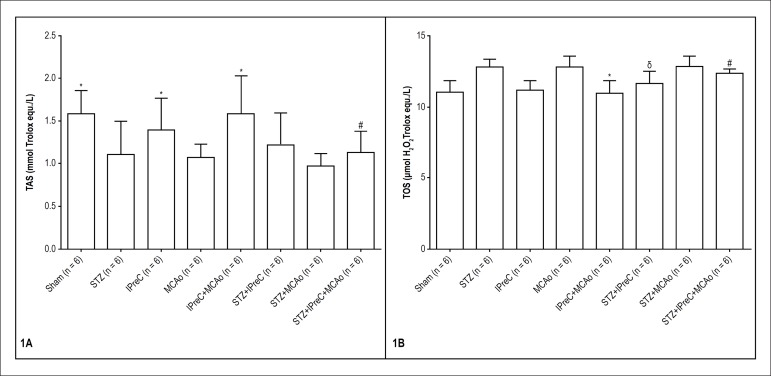
Mean myocardial TAS (Total antioxidant status) and TOS (Total oxidant status) levels in all groups. (*p < 0.05 vs. MCAo group, **#**p < 0.05 vs. STZ+ MCAo group, **δ**p < 0.05 vs. STZ group. One-way ANOVA, post-hoc Tukey’s HSD test. (STZ, Streptozotocin-induced diabetic; IPreC, ischemic preconditioning; MCAo, middle cerebral artery occlusion)

**Figure 2:**
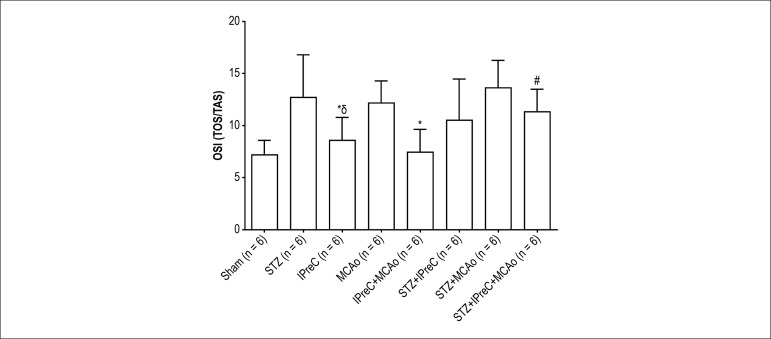
Mean OSI (Oxidative stress index) values in all groups. (*p < 0.05 vs. MCAo group, #p < 0.05 vs. STZ+ MCAo group, δp < 0.05 vs. STZ group. One-way ANOVA, post-hoc Tukey’s HSD test. (STZ, Streptozotocin-induced diabetic; IPreC, ischemic preconditioning; MCAo, middle cerebral artery occlusion)

Mean levels of myocardial total antioxidant status (TAS, Figure 1A), total oxidant status (TOS, Figure 1B) and calculated oxidative stress index (OSI: TOS/TAS, Figure 2) for all groups is shown in bar charts.

### Histopathology of myocardium

**Figure 3:**
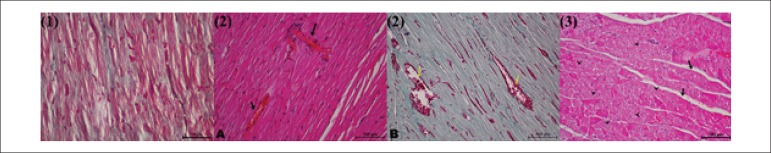
(1) Normal histological view of cardiac myofibrils in IPreC group. (Longitudinal section, Masson Trichrome stain, Magnification: 40X). (2) Congestion in IPreC+ MCAo (A) and STZ+ IPreC+ MCAo (B) groups. Overview of myofibril is nearly normal appearance. (Longitudinal section, A. H-E stain, B. Masson Trichrome stain, Magnification: 20X). (3) Eosinophilic stained areas show necrotic myofibrils that are lack of nuclei (arrow-heads) in STZ group. Arrows indicate that spaces between cardiac myofibrils. These spaces indicate necrotic area, and probably interstitial edema. (STZ, Streptozotocin-induced diabetic; IPreC, ischemic preconditioning; MCAo, middle cerebral artery occlusion, Transverse section, H-E stain, Magnification: 20X).

Histologic architecture of cardiac tissues of IPreC group was similar to that of the sham group (Figure 3(1)). Cardiac tissues of ischemic and diabetic groups showed severe histopathological alterations including congestion, necrosis and mononuclear cell infiltration. Mean congestion score (MCS) of myocardial tissue were similar between STZ and MCAo groups (p > 0.05, 2.00 ± 1.42, 2.00 ± 1.26, respectively). The highest mean myocardial congestion value was scored in STZ+ MCAo group (2.50 ± 1.10). Remote IPreC decreased myocardial congestion score in IPreC+ MCAo (1.85 ± 0.76) (Figure 3 (2a)) and STZ+ IPreC+ MCAo (1.87 ± 1.78) (Figure 3 (2b)) groups when compared to MCAo and STZ+MCAo groups, respectively. Those of differences were not found statistically significant among groups (p > 0.05).

**Figure 4:**
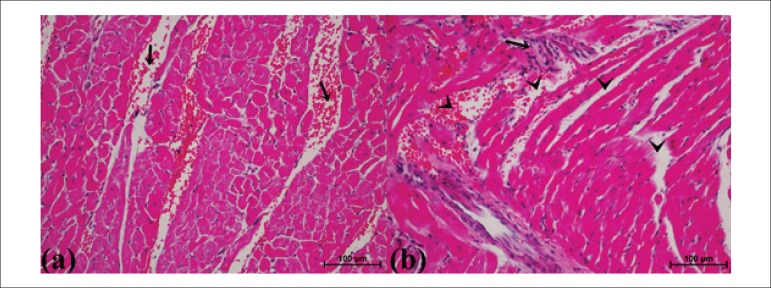
(a) Congestion (arrows) destroyed myofibrils in STZ+ MCAo group. Cardiac myofibrils are separated from each other along with congestive area. These areas probably seem to be necrotic areas accompanied by interstitial edema. (Transverse section, H-E stain, Magnification: 20X). (b) Mononuclear cell infiltration, congestion and interstitial edema in necrotic areas (arrows) destroyed cardiac myofibrils in STZ+ MCAo group (STZ, Streptozotocin-induced diabetic; IPreC, ischemic preconditioning; MCAo, middle cerebral artery occlusion, Longitudinal section, H-E stain, Magnification: 20X).

Necrotic fibrils rarely appeared in myocardial tissue of IPreC and sham groups. Mean necrosis score (MNS) of myocardium in IPreC group was recorded as 2.00 ± 1.26. That score was not found significantly different from that of the sham group (p > 0.05). Diabetes and ischemia caused extensive coagulative necrosis throughout cardiac parenchyma, in comparison with sham group (p = 0.001, p < 0.001, respectively). Following ischemia-reperfusion injury in diabetic rats, the myocardial fibrils and the nuclei of myocytes became poorly visible, almost disappeared (Figure 3 (3)) and spaces between myocardial fibrils became larger probably indicating interstitial edema (Figure 4 (b)). The highest MNS was detected as 2.66 ± 1.04 in STZ+ MCAo group. Again, there are consistent differences between the IPreC+ MCAo and STZ+ IPreC+ MCAo groups and the MCAo and STZ+ MCAo groups regarding the mean necrosis score (p < 0.001, for both).

**Figure 5:**
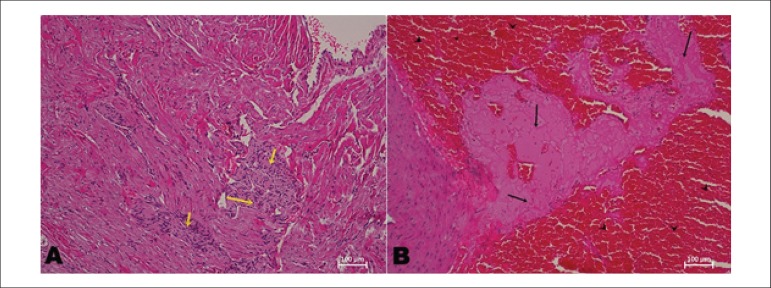
A) Degenerated myocardium caused by mononuclear cell infiltration (arrows) in MCAo group. B) Severely myodegeneration with interstitial edema, necrosis (arrows) and congestion (arrow-heads), in STZ+ MCAo group. (STZ, Streptozotocin-induced diabetic; IPreC, ischemic preconditioning; MCAo, middle cerebral artery occlusion, Longitudinal section, H-E stain, Magnification: 10X)

Mononuclear cell infiltration was mostly observed around the necrotic areas accompanied by interstitial edema in ischemic groups including MCAo and STZ+ MCAo groups (Figure 4 (b)). The highest mean infiltration scores (MIS) were recorded in MCAo and STZ+ MCAo group. Infiltration severely destroyed myofibrils and degenerated myocardium in MCAo (Figure 5A) and STZ+ MCAo groups (Figure 5B). Remote ischemic preconditioning reduced mononuclear cell infiltration in the myocardial parenchyma after MCAo in diabetic and non-diabetic rats compared with non-preconditioned controls (MCAo (2.16 ± 1.5) vs. IPreC+ MCAo (0.57 ± 0.46), p = .013 and STZ+ MCAo (2.83 ± 0.82) vs. STZ+ IPreC+ MCAo (1.57 ± 1.06), p < .001). Among STZ, IPreC and STZ+ IPreC groups, there was no significant difference regarding MIS (p > 0.05).

As a result, inducing remote ischemic preconditioning stimuli below the damage threshold, could suppress cellular inflammatory and oxidative response to ischemic injury and diabetes, which could prevent remote cardiac injury.

**Figure 6:**
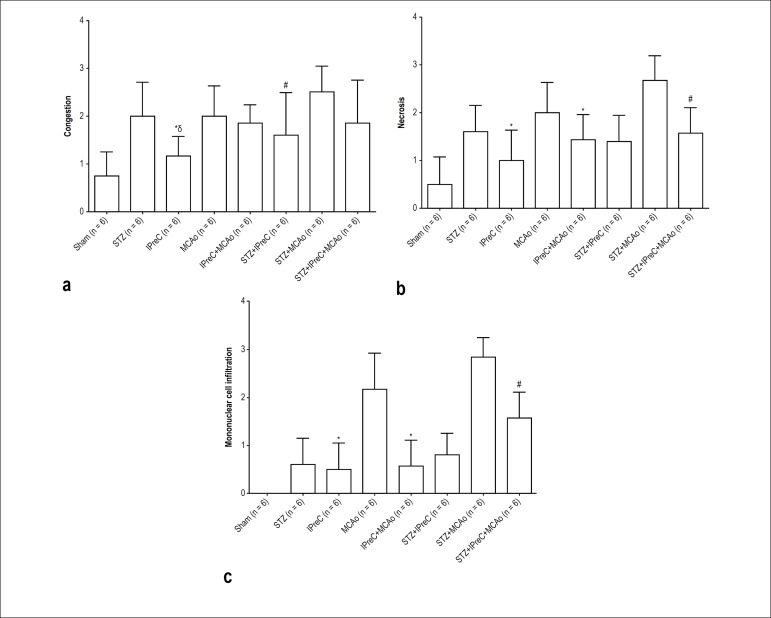
A) Mean congestion scores in all groups. (*p < 0.05 vs. MCAo group, #p < 0.05 vs. STZ+ MCAo group, δp < 0.05 vs. STZ group. B) Mean necrosis scores in all groups. (*p < 0.05 vs. MCAo group, #p < 0.05 vs. STZ+ MCAo group. C) Mean mononuclear cell infiltration scores in all groups. (*p < 0.05 vs. MCAo group, #p < 0.05 vs. STZ+ MCAo group (One-way ANOVA, post-hoc Tukey’s HSD test. STZ, Streptozotocin-induced diabetic; IPreC, ischemic preconditioning; MCAo, middle cerebral artery occlusion)

Myocardial injury scores as mean congestion score, mean necrosis score, and mean mononuclear cell infiltration score for all study groups is shown in Figure 6(a), 6 (b) and 6 (c), respectively.

### Western Blot analysis

In MCAo group, Bax/Bcl2 ratio was 1.18 ± 0.26, and that ratio was found as 0.72 ± 0.3 in IPreC group (p = 0.026) and 0.09 ± 0.06 in sham group (p < 0.001). Bax/Bcl2 ratio markedly reduced in IPreC+ MCAo group compared to MCAo group (p < 0.001).

Caspase 3 level was higher in MCAo (1.29 ± 0.12) group in comparison with both sham (0.35 ± 0.06) and IPreC (0.82 ± 0.30) groups (p < 0.001). Therefore, that level was lower in IPreC+ MCAo group (0.52 ± 0.02, p < 0.001). IPreC suppressed apoptosis progress in non-diabetic ischemic rats.

**Figure 7:**
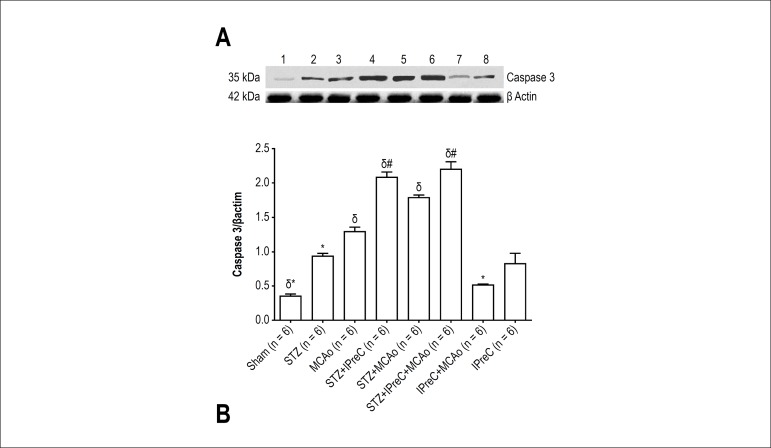
A) Western blot analysis of Bax and Bcl 2 proteins in cardiac tissues of all groups. B) Mean ratio of Bax/Bcl 2 levels in all groups. (*p < 0.05 vs. MCAo group, #p < 0.05 vs. STZ+ MCAo group, δp < 0.05 vs. STZ group. One way ANOVA, post-hoc Tukey’s HSD test. (STZ, Streptozotocin-induced diabetic; IPreC, ischemic preconditioning; MCAo, middle cerebral artery occlusion).

**Figure 8:**
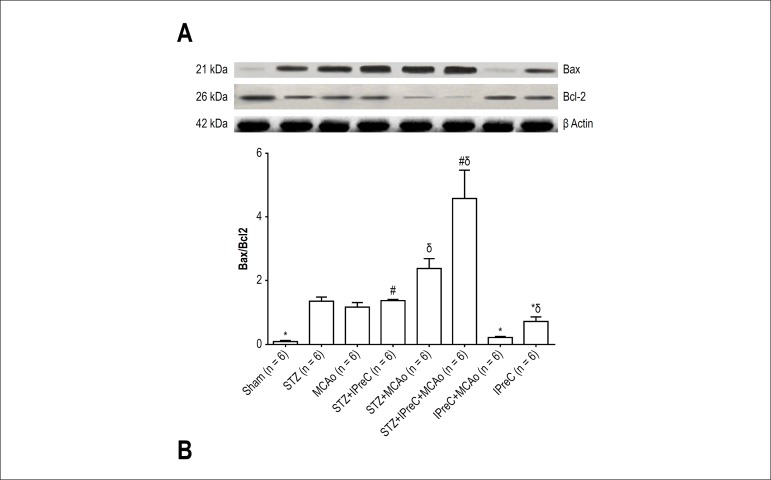
**A.** Western blot analysis of Caspase 3 protein in cardiac tissues of all groups. **B.** Mean Caspase 3 levels in all groups. (*p < 0.05 vs. MCAo group, #p < 0.05 vs. STZ+ MCAo group, δp < 0.05 vs. STZ group. One-way ANOVA, post-hoc Tukey’s HSD test. (STZ, Streptozotocin-induced diabetic; IPreC, ischemic preconditioning; MCAo, middle cerebral artery occlusion)

Myocardial apoptosis was severely induced by diabetes in all diabetic study groups including STZ, STZ+ IPreC, STZ+ MCAo and STZ+ IPreC+ MCAo groups. The highest Bax/Bcl2 ratio and caspase 3 protein levels were detected in those groups. Diabetes alone mostly induced apoptotic cell death via activated Bax and Caspase-3 proteins, and suppressed Bcl-2 activity. Preconditioning did not show any protective effect against apoptosis in diabetic groups. Western blots analysis and mean Bax/Bcl-2 ratio and caspase 3 levels of all groups were shown in Figure 7 and 8, respectively.

### Ischemic preconditioning reduces the total infarct volume

We did not observe any infarct area on the TTC-stained brain sections of STZ, Sham, IPreC and STZ+ IPreC groups. Ischemic preconditioning before cerebral ischemia significantly reduced infarction size compared with the other groups [IPreC+ MCAo (27.26 ± 20.04 mm^3^) vs. MCAo (109.07 ± 30.56 mm^3^) p < 0.001; STZ+ IPreC+ MCAo (38.70 ± 19.18 mm^3^) vs. STZ+ MCAo (165.87 ± 82 mm^3^) p < 0.001, respectively]. Also, we detected that ischemic preconditioning could improve the ischemic injury in diabetes [STZ+ IPreC+ MCAo vs. MCAo p < 0.001].

## Discussion

Cardiovascular disorders including hypertension, cardiac arrhythmias, release of biomarkers of cardiac injury, and left ventricular dysfunction are mostly seen following many types of brain injury such as trauma, ischemic stroke, and subarachnoid hemorrhage.^14,26,27^ Neurogenic cardiac injury increases risk of mortality and morbidity.^28,29^ Neurological injury affects cardiac tissue via catecholamine release and inflammation. Both of them cause cardiac cell death.^30^ Diabetes is an important modifiable risk factor for stroke, especially ischemic strokes. It was stated in previous studies that hyperglycemia also caused cardiomyopathy and resulted in similar cardiovascular complications with ischemia.^7,8,12^

Early response of myocardial cells against hyperglycemia is cardiac cell death.^13^ Two types of cell death including necrosis and apoptosis are detected in cardiomyocytes of diabetic animals.^31^ Necrosis is an uncontrolled cell death in response to oxidative stress. It causes ATP depletion and rapidly changes plasma membrane integrity, which accompanies inflammation and seriously damages not only related cells but also neighboring cells.^32,33^ In current study, we tried to evaluate remote cardiac injury following middle cerebral occlusion in diabetic rats. We observed extensive necrotic areas on myocardium of STZ and MCAo groups, especially in STZ+ MCAo groups. Moreover, inflammation and edema were seen around the necrotic areas in those groups. The highest mean necrotic score was detected in STZ+ MCAo group compared to other groups (p < 0.05). IPreC is effective in reducing MCAo-induced cardiac damage by suppressing necrotic cell death. The lower scores were recorded in remote ischemic preconditioning groups. However, mean necrosis scores of STZ and STZ+ IPreC groups were nearly similar with each other. We could report that remote ischemic preconditioning decreases the rate of necrosis induced by ischemia, even though inducing of diabetes abolish the protective effect.

The possible explanation for the result might be activation of antioxidative defense system. Oxidative stress is a primary determiner of cerebral and myocardial injuries during cerebral and myocardial ischemia/reperfusion in patients with diabetes mellitus.^11,12^ Diabetics and STZ induced-experimental animal models exhibit high oxidative stress due to b-cell dysfunction resulted in glucose toxicity; hence the activity of the antioxidant defense system is damaged by diabetes.^11,34^ TAS, TOS, and OSI are widely used in studies to determine oxidative stress activity. TOS indicates the concentration of all free oxidant radicals caused by diabetes and ischemia against to oxidative damage. Conversely, TAS is an important marker to determine the activities of antioxidant defense system against cell damage.^5^ In the current study, TOS levels were severely higher in MCAo and STZ-induced groups rather than sham and IPreC groups, whereas the lowest TAS levels were detected in those groups (p<0.001). We observed that OSI was higher in diabetic and ischemic groups compared to IPreC groups (p < 0.001). IPreC inhibited oxidative stress in both diabetic and non-diabetic rats. Myocardial TAS markedly was activated by preconditioning in diabetic and ischemic groups.

Histopathological scores of other parameters including congestion and cell infiltration also decreased in IPreC+ MCAo and STZ+ IPreC+ MCAo groups. IPreC improved histopathological alterations in diabetic and ischemic groups.

In contrast to necrosis, apoptosis is a physiologically programmed cell death; removes only damaged cells without provoking inflammation and damaging other neighboring cells.^33,35^ Experimental and human based studies showed that myocardial apoptosis increases both diabetes and STZ-induced diabetes.^36-38^ Thus, cardiac myocytes of diabetic myocardium are more vulnerable to apoptosis than non-diabetics.^37-39^Apoptosis of cardiac myocytes is commonly seen in various cardiovascular disease including diabetic cardiomyopathy, myocardial infarction, ischemia/reperfusion injury.^36^ Preconditioning is a protective mechanism against ischemia/reperfusion-induced organ injury. Ischemic preconditioning markedly reduces DNA fragmentation and apoptotic cell death in myocytes.^31^ Balance on pro-apoptotic Bax and anti-apoptotic Bcl-2 proteins regulate the mitochondrial cell death pathway.^39-41^ Bcl-2 is important in cell survival via suppressing apoptotic cell death and protects cardiac myocytes against various stress factors. Conversely, Bax is activated by oxidative stress and overexpression of Bax protein causes apoptotic cell death.^42^ Caspase-3 plays a pivotal role in the execution of apoptosis, activation of caspase-3 alone was sufficient to cause cell death in cardiac muscle.^43^ Caspase-3 activation is mostly involved in hyperglycemia induced apoptotic cell death in the myocardium.^11^ IPreC inhibits apoptosis by altering balance between pro-apoptotic and anti-apoptotic proteins, and inhibiting caspase activity.^44,45^ In our study, we found that remote ischemic preconditioning decreased the Bax/Bcl2 ratio and caspase 3 activity following ischemia-reperfusion injury (IPreC+ MCAo vs. MCAo group, p < 0.001). Hence, myocardial injury score in STZ-induced diabetic rats was detected as high as MCAo rats. Unlikely non-diabetic rats, remote IPreC did not play a role on inhibiting the apoptotic cell death in diabetic rats.

## Conclusion

As a conclusion, cerebral ischemic preconditioning attenuates myocardial injury via ameliorating histological findings, and activating antioxidant mechanism, and inducing anti-apoptotic activity in and diabetic rats. Preconditioning has also anti-apoptotic effect in non-diabetic rats, whereas it has not same effect in diabetic rats. We could suggest that the reason is that preconditioning process was applied after the Streptozotocin injection, thus apoptosis was already induced by diabetes before preconditioning process. As a result, we could assume that remote IPreC might not be shown protective effect against apoptosis in diabetic rats. Further experimental studies could be done to determine possible mechanisms that may explain the loss of ischemic preconditioning in diabetic hearts; vascular and biochemical changes on myocardium associated with STZ-induced hyperglycemia could be evaluated at different time intervals after remote ischemic preconditioning.
